# A Model for Effective Learning in Competition: A Pedagogical Tool to Enhance Enjoyment and Perceptions of Competency in Physical Education Lessons for Young Children

**DOI:** 10.3390/children11010111

**Published:** 2024-01-17

**Authors:** Neil Castle, Kristy Howells

**Affiliations:** Department of Sport, Exercise and Rehabilitation Sciences, School of Psychology and Life Sciences, Canterbury Christ Church University, Canterbury CT1 1QU, UK; kristy.howells@canterbury.ac.uk

**Keywords:** competition, early years, physical education, enjoyment, perceived competence

## Abstract

To date, little research on competition has focused on young children (6–7-year-olds). A total of ninety-seven participants (51 boys and 46 girls) from two English primary schools completed two physical education (PE) lessons, which included three different activity challenges. The control group undertook the same activities in both lessons. The experimental group did likewise but were set high-, low-, or mid-level targets in lesson two based on individual scores from lesson one. The children completed a post-session questionnaire to assess (i) enjoyment levels and (ii) which activity they perceived they performed best in. The results found that children both improved and enjoyed the lesson most when low- or mid-level targets were set. Indeed, when targets were absent (in the control group), children’s competency scores regressed. Likewise, children perceived that they performed best in the activity where lower targets were set. Their perceived competency included both tangible and intangible reasons. From these results, it is recommended that for practitioners working with 6–7-year-old children, the most effective learning in competition uses individualised and competitive targets and challenges as a means to garner greater enjoyment in PE. Understanding each child’s self-efficacy and motivation is key, which requires ongoing evaluation and assessment during PE lessons.

## 1. Introduction

To date, little research on the enjoyment of competition has focused on young children (6–7-year-olds), particularly in the UK, yet this age phase is a key time for habits of likes and dislikes to be formed [1]. However, there is some evidence to suggest that children are aware of the concept of competition and that they can exhibit competitive behaviors from the age of four [2]. Enjoyment has been identified as being crucial in determining the willingness of children to participate in physical activity [3], and it has been emphasised how little research has been undertaken in this area on primary-aged children [4]. Much of the current research [5] focuses on competitive sport that takes place outside of curriculum time and considers the attitudes of older children, highlighting the need to focus more on PE lessons and younger pupils. Yet, competition sits prominently within the English National Curriculum for Physical Education (PE) [6], with young children needing to ‘engage in competitive (both against self and against others) and co-operative physical activities’ [6] (p. 2), which then develops in the curriculum documents as the child ages to ‘enjoying competing and collaborating’ [6] (p. 3). Yet, there is little guidance for teachers on how competition should be delivered.

### 1.1. Competition in the Curriculum

Competition (and competitive sport) has been a central tenet of English PE policy and practice for many years, reflecting the political philosophy of the government at the time of the last curriculum reform in 2013 [7]. However, many cite the negative consequences of competition, particularly for the youngest and most vulnerable children, as a source of lowering self-esteem, confidence, and competence that can drive lower disengagement that reduce the chances of a lifelong passion for health and well-being [5].

On the one hand, competition can be seen as an effective tool to help develop skills, encourage physical activity, build character, appreciate and respect others, and prepare children for ‘real life’ [8,9,10]. PE lessons throughout the years have been seen as the ideal setting for allowing children to explore how to cope with winning and losing to develop good sporting behaviours [11]. Yet, Richardson [12] found that two-thirds of children aged eight to sixteen reacted badly when they lost and, moreover, their parents also behaved badly when watching their children lose. However, others suggest that adopting a more moderate approach that focuses more on cooperation is wrong, proposing ‘remove the competition and you remove the fun’ [13] (p. 11). Moreover, Howells [14] stresses the importance of practitioners taking care when planning competitive activities within PE lessons, citing guidance from the House of Commons Education Committee [15], which suggests that, if poorly delivered, competitive activities can deter children from engaging in physical activity and sport in the future. Competition is perceived to be a vehicle by which appropriate levels of challenge can be delivered [16]. In order to make competitive experiences meaningful, teachers should ensure that when delivering competition, the ‘emphasis be placed on the challenge(s) inherent in the process of competing rather than on the outcome (that is, winning and losing)’ [17] (p. 302).

Other countries may not be quite so implicit in their advocacy of competition as a focus for PE lessons, but a recent comparison of the PE curriculums from 27 EU countries suggests that ‘sport education’ is a key concept (instead of explicitly competition) that still underpins PE in many of these countries. Such curriculums seek similar outcomes developed around performance, tactical awareness, collaboration, fair play, and the learning experiences that come from winning and losing. Moreover, the authors also suggest that future reforms in Italy and France are aimed at ‘creating a positive attitude’ towards participation in competition [18] (p. 7332).

In Ireland, the PE curriculum document makes specific reference to the role that ‘a balanced approach to competition’ can make, arguing that competition ‘is not incompatible’ with the broader holistic aims of PE and ‘can make a significant contribution to the child’s development while at the same time providing fun, enjoyment and satisfaction’ [19].

In Canada, where the PE curriculum places a high focus on children developing physical literacy, one of the key learning standards outlines that children are expected to ‘identify and describe preferred types of physical activity’, which includes ‘individual activities or activities with others’, and ‘competitive or non-competitive activities’ [20]. Likewise, the USA National Standard 5 for physical education states that ‘the physically literate individual recognizes the value of physical activity for health, enjoyment, challenge, self-expression and/or social interaction’ [21].

The English national curriculum for PE provides a minimalist outline of key aims and objectives. The purpose of this study is to describe how the most effective PE curriculum will inspire children to ‘succeed and excel in competitive sport’ [6] (p. 1). However, as with several of the other countries mentioned, little guidance and pedagogical support is offered as to how the aims can effectively be achieved, let alone how competition should be effectively taught [7]. The individual philosophies of practitioners often influence what is delivered in PE lessons and how it is taught. This can lead to a lack of congruency between what the PE curriculum guides staff to deliver and what actually takes place in PE lessons [22]. Therefore, it is important to examine the application of pedagogical strategies designed to support enjoyment and perceived competency in order to potentially aid the teachers of the future.

This study was designed to investigate the practical application of a model that has been designed to address some of the challenges posed by the limited guidance offered specifically within the national curriculum for PE in England. The future implications from these initial findings may also be useful in providing support for teachers in England and other countries who want to consider how enjoyment in PE can be fostered through challenges with and alongside others.

### 1.2. Competition in the Early Years

There is limited research in the field of competition in the early years, and where research can be found, opinions differ significantly, highlighting the importance of this study, as it brings new information to the field. Children aged 3–7 are at the pre-operational stage [5], which is also referred to as the pre-logical phase of development and, as such, children are still quite egocentric [23]. Consequently, at this age phase, they may struggle to work socially and emotionally with others, particularly in competitive scenarios. It is recommended that teachers should focus on helping these young children to master fundamental movement skills (FMS), and competition should take the form of individual challenges to achieve personal bests [5]. This echoes Ofsted’s [24] recent proposal that competition can be most effective when teachers provide ‘varying degrees of challenge,’ and that competition can be effectively delivered via individual challenges against oneself (rather than necessarily against others). If children have yet to achieve mastery in skills, they will, therefore, lack competence. These children may struggle to compete against others, and if placed into competitive situations too early, it ultimately may have a detrimental effect on their confidence to engage in the future [24].

Tsiakara and Digeldis [25] previously recommended that teachers should avoid setting competitive goals for younger children at all so as to avoid negatively impacting motivation when children fail to achieve goals or make unfavourable comparisons to their peers. They suggest this even though they found that children as young as four understand the concept of competition and can exhibit competitive behaviour through the motivation to succeed. Working with pre-school children throwing bean bags into hoops, they found that the children performed better when setting competitive targets rather than simply being asked to do their best.

In presenting the Model for Effective Learning in Competition (MELC), Howells et al. (2018) [26] consider how competition can be delivered effectively in primary school PE lessons. In doing so, they present three different types of competition that teachers may consider when planning their PE lessons [26]: competition against, alongside, and with others. For children who have a perceived lack of competence, competition in PE lessons that pitches individuals against each other with winners and losers, or whereby they find their scores ranked in comparison to others, is demotivating and can have negative affective outcomes [27] that could impact future engagement in physical activity outside of school. Conversely, individual challenges have been associated with ‘a sense of achievement, competence and autonomous motivation’ [27] p. 8. Even children who do not normally enjoy competitive activities in PE have demonstrated more positive affective motivation because of the sense of accomplishment associated with the personal sense of achievement when they overcome individual personal challenges [28]. Consequently, for six- and seven-year-olds, where focusing on mastering FMS is considered, competition ‘alongside others’ is recommended as the most appropriate type of competition to use with this age phase [26].

Competing alongside others is when children work independently of others to improve on previous personal best scores. Others do not directly influence an individual’s performance, but working alongside their peers may motivate children to work harder [26]. Children are then encouraged to achieve, e.g., their longest distance, fastest time, or best score through demonstrating increased mastery of skills. This approach helps teachers focus on individual competency, which enables them to create differentiated targets that can have a huge impact on their perceived competency and confidence levels within PE [26]. Therefore, as this study examines the impact on children’s perceived competence and enjoyment, the activities were delivered using the competition alongside others approach.

### 1.3. The Model for Effective Learning in Competition (MELC)

**Figure 1:**
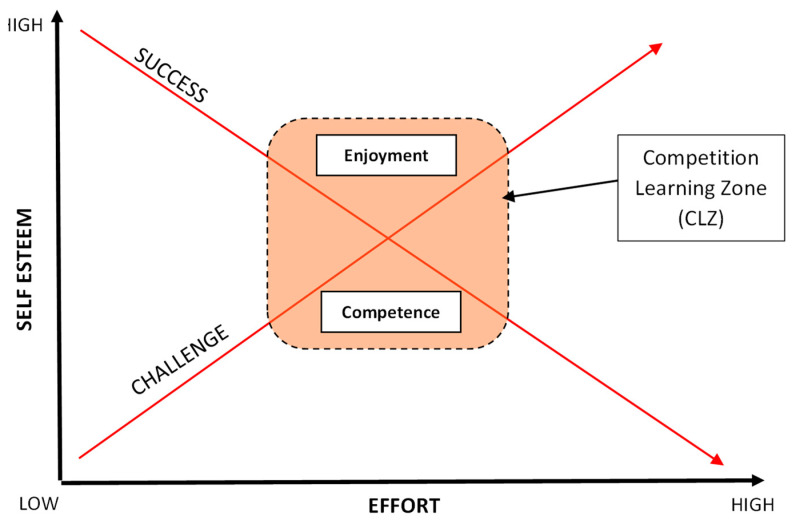
Adapted Model for Effective Learning in Competition (MELC) [26].

With such conflicting views and perspectives on what competition is and how it should be delivered effectively, particularly with younger children, it is unsurprising that primary teachers, (who are often in England not trained as PE specialists), lack the confidence to teach the subject effectively [29]. Howells et al. [26] attempt to unpick much of the confusion. Through the creation of a ‘Model for Effective learning in Competition’ (MELC), they explore the relationship between the level of challenge offered within a competitive activity and the level of success achieved. They suggest there is an optimal zone for learning when these two variables are in equity, but an individual is required to sustain a reasonable amount of effort to achieve that success. This area is called the ‘Competition Learning Zone’ (CLZ) (see Figure 1) [26]. This study examines the MELC in action to assess the impact on children’s perceived competence and enjoyment when they were set differing levels of competitive challenge and aims to help teachers understand what ‘just right’ looks like in a typical PE lesson and where this may need to be adjusted for different individuals.

This idea of an optimum area for effective competition was developed from Csikszentmihalyi’s ‘flow’ theory (2008) and aligns closely with the principle of challenge that is delivered ‘just right’ [30]. Although the MELC [26] has similarities to Csikszentmihalyi’s [31] work on flow, there are some key differences, in particular when considering how competition should be delivered within PE lessons. Whereas the flow model cautions about increased levels of anxiety and boredom when flow is not achieved, the MELC [26] proposes that effective learning can take place outside of the CLZ and may even be more beneficial to certain children. For example, Howells et al. [26] suggest that situations where success can be achieved at lower levels of challenge with reduced effort can have a positive impact of increasing children’s self-esteem. If this, in turn, creates greater perceived competency and enjoyment, then the children may be willing to apply themselves more when challenges become harder. Consequently, this approach [26] is ideal when working with children of lower ability or confidence or when children are trying to become competent in a new activity. Equally, they propose that more able children can learn to become more focused and resilient by undertaking challenges that are increasingly harder and require even more sustained effort but do not always achieve success. Certainly, if managed correctly, creating these environments may support children learning to cope with hardship and develop resilience.

### 1.4. Enjoyment

A big factor in children’s enjoyment of PE is their level of competence, to the extent that it has been suggested that it is the single factor most likely to determine their participation in PE lessons [16]. The impact of enjoyment in PE lessons has been explored [32] by considering not only how children define fun and enjoyment as separate constructs but also how these impact their attitudes towards PE across different age groups. They argue that whilst enjoyment is often valued in developing positive attitudes, fun has not always been perceived as an ‘appropriate outcome’ [32] (p. 3) in PE lessons. However, ‘a lack of fun can have a deleterious effect on participation and meaningfulness of an experience’ [17] (p. 300). Equally, Rikard and Banville’s [33] research highlighted how activities that were not perceived as being fun were a major factor in children choosing not to take part in lessons. One of the recommended steps to effectively deliver ‘true competition’ is to ‘aim for enjoyment’ [9] (p. 10). They suggest several ways that this can be achieved, including the importance of setting challenging but achievable tasks. When tasks are too easy, children will become bored and likewise, they will quickly become frustrated with tasks that are too difficult. DfE [6] also stresses the importance of enjoying competing within the National Curriculum.

A recent study sought to engage with primary-aged children in Finland to develop a greater understanding of what drove children’s enjoyment of PE lessons using achievement goal theory as the framework for their research [3]. Results from the sample of 1148 pupils (mean age = 11.27) confirmed previous research, suggesting that task-involving climates (environments where the emphasis is placed on personal development and improvement that comes from individual effort to overcome personal challenges) produced positive associations with enjoyment. Conversely, ego-involving climates (environments where social and normative comparisons are emphasised, often through competition against others) produced a direct negative association with enjoyment (along with raised anxiety) that may disengage children from future physical activity [3].

Consequently, it could be argued that competition that delivers appropriate levels of individual challenge could positively impact levels of enjoyment in PE lessons and potentially children’s ability to experience a state of ‘flow’ [31]. Likewise, children who enjoy their PE lessons are likely to be more active and engaged, and their capacity for developing greater competence and confidence increases. From these findings, it would appear that competition ‘alongside others’ [26] would be the most effective pedagogical approach to use in this study.

Based on the review of the literature outlined within the introduction, the purpose of this study was twofold: to examine young children’s enjoyment and perceived competency levels of competing alongside others, (whereby they seek to overcome individual challenges based on personal best scores) and to specifically examine how the pedagogical strategy of the MELC [26] could be used to support children’s enjoyment and perceived competency in competition. The MELC explores the relationship between the level of challenge within an activity and the level of success achieved, suggesting that there is a ‘Competition Learning Zone’ (CLZ) when these two are in equity. When children are working in the CLZ, success provides positive experiences and helps develop perceived competency and enjoyment [26].

### 1.5. Competence

Perceived competency is an often under-researched topic in young children due to their sporadic nature of physical activity. It has been noted previously that young children, when asked to estimate their physical activity levels, underestimate their perceived levels [34]. D’Hondt et al. [35] also highlight differences between perceived levels and actual levels of young children’s aquatic skills, emphasising that perhaps children have difficulty perceiving their levels of competency in motor skills. Yet, within the National Curriculum for PE [4], young children aged 5–7 years are expected to become ‘increasingly more competent and confident’ and master basic skills (p. 2). Self-determination theory [27] identifies competence as one of three basic needs that all humans seek. The idea that humans need to develop competence to achieve mastery of tasks that they perceive are important to them is key to them being motivated to act. Moreover, Bailey [36] argues that physical competence can be a significant factor that drives social acceptance in children, and by doing so also develops ‘their personal confidence and self-esteem’ [37] (p. 5). Goodway et al. [23] provide valuable context for this by suggesting that competence is described as being the point at which children are mechanically efficient, coordinated, and controlled in their movement patterns when performing fundamental movement skills (FMS) in isolation (initially) and in combination with others. One of the six features that is fundamental to creating meaningful PE lessons is the concept of motor competence; i.e., when children feel they have learned new skills and perceive themselves as being more motor competent [17]. Conversely, they acknowledge research by Erhorn [38], where interviews and observations of primary school-aged children concluded that low levels of perceived competence were linked to lower levels of enjoyment in PE and increased chances of children not participating satisfactorily in the lesson.

## 2. Materials and Methods

### 2.1. Context

The research was set up as a bespoke experimental design research project that focused on the same targeted socio-economic and geographical areas to allow for comparisons between the two school settings due to the contextual similarities of the settings. This research aimed to examine if the MELC pedagogical strategy can develop enjoyment and perceptions of competency through the use of target setting. The schools were purposefully chosen [39], as they were recognised locally for their provision of PE and their commitment to school sports. Both the PE coordinators in charge of leading PE within the schools were trained specialists, and both schools had been used within case studies as examples of best practices within the region [26].

### 2.2. Participants and Their Schools

Each of the two schools had above-average numbers of children on roll. School A had 428 children and School B had 283, and the UK national average was 281 [40]. The combined number of children in the research across both schools was 97, and all were in Year 2 (aged 6 or 7 years). Four classes in total were used, and each school had one competition (experimental) group and one control group. There were 50 children in total in the competition group and 47 children made up the control group. Neither school used any selection criteria based on academic or physical ability. The two classes in each school were heterogeneous in nature [41].

An experimental design was used in which one class was the control group and one was the competition group. The school chose the groups. Both classes in each school undertook two PE lessons (sessions 1 and 2). In those lessons, they rotated around three different activity ‘stations’: running, jumping, and throwing and catching (see Table 1). Session 1 generated baseline scores for each pupil in each activity. In session 2, the control groups repeated the first session, and new scores were collected. The competition groups also repeated the same activities; however, this time, every child in those groups was given a specific target for each activity based on their individual scores from session 1. In the running activity, the pupils were set a low target that was 10% less than their score in session 1. In the jumping activity, the children were set a high target that was 10% greater than their individual result from session 1. Finally, for the throwing and catching activity, the children were set a target in session 2 that was equal to (or no change) from their score in session 1. This was classified as a mid-level target (see Table 2). The purpose of this study was to assess differences between the two groups in terms of their level of competence (measured through improvements or regressions in their running, jumping, and throwing and catching scores). Also, their enjoyment of the activities across the two sessions was evaluated (through a short questionnaire distributed after session 2) in order to evaluate the impact on the competition group of the individualised targets that were set for them.

There were a number of children who only attended one of the two sessions (not included in the data collection numbers above). Although they were allowed to participate in the activities, only data collected from children who attended both sessions were used in this research.

### 2.3. Ethical Considerations

All data collected were pseudonymised and stored via password-protected mechanisms that were only accessible to the researchers and were in line with the University-set GDPR protocol. All paper questionnaires were destroyed once they had been logged into an Excel spreadsheet. All participants were given the option to withdraw from the study at any time without giving a reason, and their results were removed. Personal demographic details of sex were asked and recorded to support analysis. Ethical approval was granted from the Canterbury Christ Church University Research Ethics Committee (17/EDU/010, approved 15 December 2017) and the ‘gatekeepers’, the head teachers of each school, as the activities were curriculum-based running, jumping, and throwing and catching skills that are undertaken within normal PE lessons. Children are also commonly asked about likes and dislikes; therefore, the head teachers deemed the project age appropriate and common practice within the educational setting and age phase and gave their consent loco parentis. Children’s assent was gained by explaining the activities to the children at the start of each class. To prevent bias, all responses and scores for children who attended both lessons were included in the data analysis, and only questionnaires that had been fully completed were included. A short time frame of data collection was implemented to examine current feelings and restrict other external factors impacting perceived competency and enjoyment levels.

### 2.4. The Activity Challenges

This study required all participants to be physically able to complete the challenges set for them. This was mitigated through careful selection of the tasks and purposeful timing of when the assessments took place. The activities selected for the research were developed by the organisation ‘Fit for Sport’ [42] as part of an Activity Challenge programme that was delivered to over 10,000 primary-age children [42]. These activities have been created specifically for school children, in conjunction with the National Curriculum end-of-age phase targets [6], and over 10,000 children have used the activity challenges, providing a level of validity that was considered extremely important for this research. The tests also offer reliable test–re-test data, as they are very simple to set up and have very clear instructions, limited equipment is required, and the activity challenges are very easy to score, making these activity challenges very age appropriate (see Table 1).

The activities have been developed around running, jumping, and throwing and are considered to be three of the basic fundamental movement skills that underpin all sporting activities [23]. One of the four aims of the PE curriculum in England is for all children to ‘develop competence to excel in a broad range of physical activities’ [6] (p. 1). Additionally, these three skills are specifically mentioned in the subject content at Key Stage 1 (ages 5–7 years).

Children should be taught to:

- Master basic movements, including running, jumping, throwing, and catching [6].

This research took place purposefully in the final few weeks of the school year in the summer term, as the participants were coming to the end of the Key Stage 1 phase of their education. Therefore, at the point where the skills were being researched and assessed, they were familiar to all the children, and they should have been mastered. This level of mastery was confirmed by the staff at each school, ensuring internal consistency.

A number of strategies were introduced to give the process high levels of internal validity. The equipment and working areas used within the PE lessons were replicated across both schools to ensure consistency in the delivery of the activities. The sessions took place in the timetabled PE lesson for each class, ensuring that session one and session two took place at the same time on the same day. Each session took place outside on the respective school playgrounds and the weather was clear, warm, and sunny, with no evident wind. The researcher ensured that the same equipment was used for all sessions and took personal responsibility for organising each station, ensuring that the distances for the running, jumping, and catching/throwing activities were carefully and strictly measured in alignment with the Fit for Sport guidelines [42].

### 2.5. Questionnaire

In order to garner an understanding of the children’s enjoyment of the activities, each child was given the opportunity to complete a short questionnaire after the completion of session 2. Bell [43] highlights the value of gathering data from young children via questionnaires rather than by proxy via adults and provides guidance on how this can be effectively achieved, considering some of the challenges of this particular audience. Children may find memory recall more challenging than adults, so in order to make sure that the questions were ‘in the here and now’ (p. 464), as recommended by Bell [43], the questionnaires were completed immediately at the end of the second session when the children were back in the classroom.

To address any potential bias prior to the questionnaire being completed, the researcher emphasised to all children that there were no right or wrong answers. Questionnaires for this age group must be simple using ‘short questions with straightforward syntax’ [43] (p. 464). With the youngest children in the research potentially still being only six years old (who may find comprehending language and reading hard), each of the questions was supported by emojis and iconographic images to represent the running, jumping, and catching/throwing activities to help the children explain their answers. There were just four questions. Question one asked them to reflect on how they felt at the end of the lesson, selecting from a set of faces showing different expressions. The children were then required to underline which activity they enjoyed most and least and which activity they felt they performed best in, and then to offer qualitative reasons to explain their answers.

Thomas [44] claims that image-based approaches to data collection provide a ‘powerful extension’ to more traditional methods, especially for younger children or those who may find it hard to engage and understand words. In this case, it ensured that children who may still be developing their reading skills could recognise a visual representation of the activity. Caution is advised [45], and ‘feelings questions’ (p. 36) must be structured in such a way that the research children are completely clear about what is being asked. This may be achieved when working with young children using closed questions [46], and this approach is particularly useful, in so much as it enables the researcher to interpret quantifiable data from the responses and qualitative responses.

To ensure internal consistency and validity of the questionnaire, the staff at each of the schools checked and approved the age appropriateness and the level of language comprehension within the questionnaire. This bespoke approach was used due to a lack of similar pre-existing questionnaires for this age phase.

### 2.6. Data Analysis

For the Fit for Sport activity challenge data collection, the number of shuttle runs, the number of star jumps, and the number of two handed bounces were collected for each session. The data were then examined in terms of improvements, regression, or remaining unchanged. This also allowed for analysis of the introduction of targets for the competition group or the lack of targets for the control group. Hope [46] suggests that percentages are the most appropriate measure to use when comparing ‘between 35 and 100 subjects’ (participants) [46] (p. 62). Thus, to evaluate the impact of individual targets, scores were calculated for the percentage of children in each group whose scores in session 2 either improved, regressed, or remained unchanged. Inferential statistics were also completed to compare the results for the two groups, and a Mann–Whitney U test was undertaken within SPSS 26.0 statistical analysis (IBM Corp, Armonk, NY, USA) to assess the group differences. Statistical significance was set at <0.05.

For the questionnaire data collection, the activity the children enjoyed the most and least and the qualitative reasons why they chose that activity as the most/least enjoyed were recorded. The data were then examined in terms of a thematic approach of tangible and intangible responses. This approach draws on goal setting and motivational attribution theory from sports psychology [13,47,48]. This grouping thematic approach was deemed the most appropriate, as it has been previously used to examine primary-aged children’s views and perspectives of physical education, in particular their perceptions about what ‘being good in PE’ meant to them, and the influence of enjoyment within these views [49].

## 3. Results

The results present the findings from a small-scale comparative case study that focuses on a series of sequential PE lessons delivering competition ‘alongside others’ at two schools within a very close geographic location and socio-economic context. It seeks to offer an insight into the practical application of the MELC [26], and in doing so supports teachers at these schools in considering how they can best deliver competition as a means to help improve movement behaviours and motor skills in early years. The Discussion Section will then consider how the implications of this small study may be considered for future research and potentially extended to a broader population.

### 3.1. Activity Challenge Results

**Figure 2:**
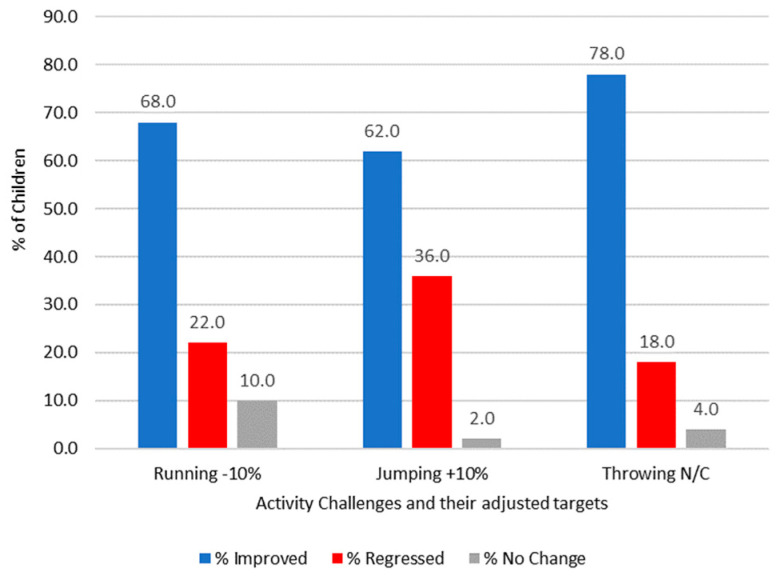
The overall percentage of children in the competition group who have improved, regressed, or not changed their activity challenge scores.

**Figure 3:**
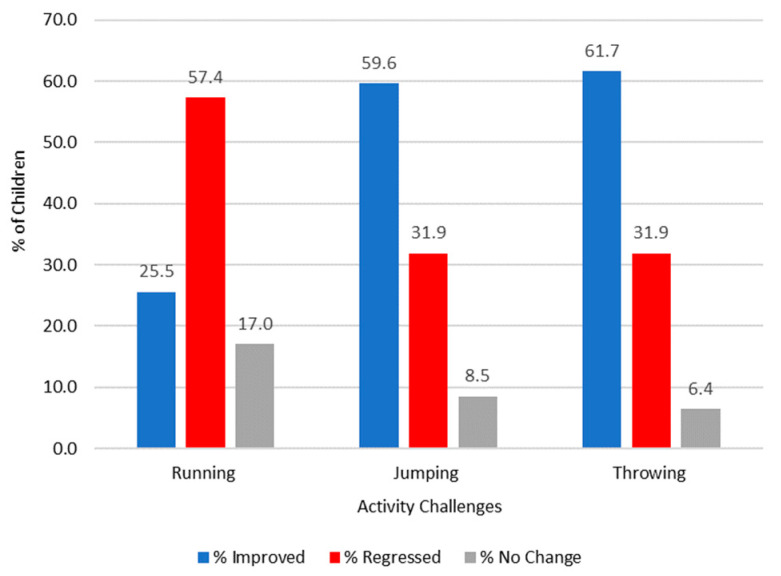
The overall percentage of children in the control group who have improved, regressed, or not changed their activity challenge scores.

When Figure 2 and Figure 3 are compared, the data indicate that young children from the assessed schools may need competition in order to stay engaged in PE lessons. A high proportion of children in the control group regressed in their performance and activity scores when they did not have a target to aim towards. It appears that the children lose focus, and competence decreases for all activities. Significant differences were found for running, with the results indicating that children were able to be more successful when targets and competition were included in the sessions; z = −3.955 and *p* ≤ 0.001. There were no significant differences found for jumping (z = −1.397, *p* = 0.162) and throwing (z = −1.708, *p* = 0.088). Therefore, adding competition and targets as a pedagogical strategy can help children master the skills they are developing within the early age phases of school. There is also a suggestion from the data that using individualised challenges bespoke for the children is a key way to ensure the progression of motor skills over consecutive PE lessons.

### 3.2. Enjoyment

#### 3.2.1. Most Enjoyed

In the competition group, where children were set individual competitive targets, there was a clear preference for enjoying the running activity most (52% n = 26) compared to the percentage of children who selected the jumping activity (22% n = 11). This was even more pronounced amongst the girls, with 65.2% (n = 15) suggesting they enjoyed the running activity most, whereas only 13% (n = 3) selected the throwing and catching activity. For these children in the competition group, the running activity posed individual targets in session 2 that were 10% lower than the children’s scores achieved in session 1, suggesting that setting children low but achievable targets may be one way to increase enjoyment in PE lessons. The viewpoints of the children for their reasons for enjoying running are illustrated in Table 3 and grouped for tangible and intangible reasons. The competition group made specific reference to improving their score or hitting and achieving their target when describing which activity they felt they scored the best in. Even though the control group did not have a target set, some children still mentioned their score as the reason why they performed best, and they were aware of their perceived placing overall in the class, commenting ‘I got the highest score in my group’ and ‘because I completed the most runs in the class’. It appears that even without targets, children value comparing scores as a means to gauge their own performance.

The control group had no clear preference for the activity they most enjoyed, and their answers were more evenly spread across the three activities, with 36% (n = 17) choosing running and 32% (n = 15) opting for both the jumping and throwing/catching activities. Both the control (38.3%, n = 18) and competition (46%, n = 23) groups selected the running activity as the activity they perceived they performed their best in, and the jumping activity as their perceived lowest score (control group 29.8%, n= 14, and competition group; 26%, n = 13).

#### 3.2.2. Least Enjoyed

A total of 52% (n = 26) of the children in the competition group picked the jumping challenge as the least enjoyable, with 26% (n = 13) selecting the catching and throwing and 20% (n = 10) selecting the running challenge. The jumping activity was the activity in which the most challenging targets were set, indicating that maybe one way to decrease enjoyment in PE lessons is by setting tasks that are perceived as too hard. The viewpoints of the children for why they least enjoyed an activity are illustrated in Table 4 and grouped for tangible and intangible reasons. Once again, there was a far more even spread of responses from the control group, with only a 6% difference between the scores for each of the three activities.

## 4. Discussion

Competition has been a significant feature of PE lessons for many years but not all pupils enjoy competition and not all children succeed in competitive environments, supporting the notion that ‘competition might form the environment of participation but not the goal: it is the medium not the message’ [50] (p. 46). This study was designed to consider how the effective use of competitive practices might positively support the development of physical competencies in all children (regardless of their level of ability) and how the environment created by different levels of competition impacts their perceived enjoyment of the subject.

This study aimed to examine how the MELC could be used as an effective pedagogical tool in terms of where the apex of the CLZ should be for young children and how this translates into target setting and competing alongside each other [26]. The findings reveal that children enjoyed most the activities that have lower targets, enabling them to be the most successful, and they also improved the most within this situation. The results highlight the need to adapt the MELC for young children and share how individualising the tool could be a useful practice in the future when teaching PE to young children.

### 4.1. Using the MELC as an Effective Pedagogical Tool

Improvements in the activity scores were evident amongst the competition group in all three activities, and the highest percentage of children improved when mid-level targets were set. There were no differences between genders with regard to the improved competence of children, which is consistent with previous findings [51,52]. The results from this study could suggest that when applying the MELC to the children, the apex of the CLZ [26] sits in and around an individual’s ‘personal best’ (where the mid-level challenge was set in the throwing activity). However, on closer scrutiny, when making direct comparisons between the number of children who improved in the competition group and the corresponding results for the same activity in the control group, the biggest difference in scores was found in the running activity where low targets were set in the competition group. A total of 68% (n = 34) in the competition group improved their running score compared to just 25.5% (n = 12) who improved in the control group. The throwing activity, where there was a mid-level target (set at their previous personal best), saw the highest percentage of children from the competition group improving their throwing score at 78% (n = 39), whilst in the control group, 61.7% (n = 29) of the children improved.

**Figure 4:**
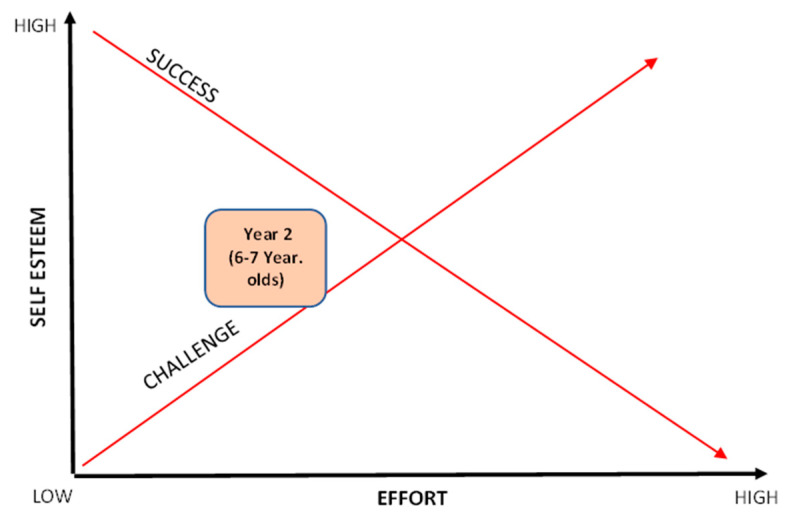
The MELC with the new CLZ position for Year 2 children (6–7 year-olds) to improve perceived competence and enjoyment in PE lessons.

It is, therefore, proposed (from the data uncovered in this research) that for the children in this study, the CLZ exists more towards the left in the MELC (Figure 4), when challenges are set just below children’s personal best scores (in this case 10% lower). Lower targets facilitate enjoyment and perception, as well as actual competency with younger children. This may be due to their stage of physical and motor development, whereby the skills within the activities are still a ‘work in progress’. Therefore, it is recommended when teaching this age phase that the CLZ is positioned as illustrated in Figure 4.

#### 4.1.1. The Impact of Competitive Targets on Enjoyment

Children’s enjoyment of the activities was influenced by the presence of competitive targets. When asked which activity the children enjoyed the most, the responses from the control group were evenly spread across all three activities. However, children in the competition group showed a clear preference for the running activity where they were set low targets, and the activity that the fewest children claimed they enjoyed most was the jumping activity, where high targets were set for them. This links to Sport England’s [53] proposal that enjoyment is the biggest driver of activity levels amongst children between the ages of five and sixteen. This may be why the National Curriculum of PE [6] specifically references that children should ‘enjoy communicating, collaborating, and competing with each other’ [6] (p. 2). The MELC [26] shows us how to set targets and use the competing alongside others approach within the young age phase.

Conversely, when asked which activity the children enjoyed least, over half of the children in the competition group chose the jumping activity (with its high targets). These results would appear to support the argument that children in this age enjoy PE lessons more when lower competitive targets that they feel are attainable are set. It could be argued, therefore, that teachers could use low competitive targets as a tool to make a less enjoyable activity more enjoyable in the future. The key may be in understanding how children perceive fun and enjoyment in PE. This is a complex challenge and, as most primary teachers are not PE specialists, ‘enjoyment and perceived competence are not always their first priority’ [4] (p. 37). Yet, this study has shown the importance of linking enjoyment to PE lessons for children to help aid children’s improvements in their performances and scores. One of the key recommendations of this study is for teachers to understand and appreciate not just that children have different levels of abilities but also differing levels of motivation and engagement.

**Figure 5:**
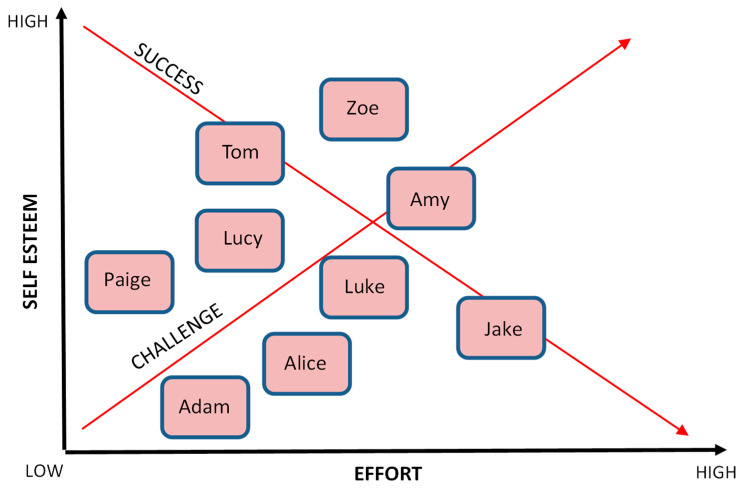
The MELC with the new individualised CLZ placements for the least and most confident children within the class (names are pseudonyms).

Creating regular opportunities for children to achieve success by completing competitive challenges (set at an appropriate level for each child) can help to raise their perceived competency and enjoyment in PE lessons. However, teachers should also seek to understand more about what drives each child’s enjoyment by gathering additional qualitative feedback to understand how each attributes their success or failures. In particular, it is recommended that teachers explore the motivations of children who are the least confident and the most confident in their classes and seek to understand how their enjoyment of the lesson can be enhanced. Whilst 36% of the children associated enjoyment with success in achieving targets set for them, 46% of the same group gave far more diverse reasons for why they ‘enjoyed’ an activity most. One boy enjoyed the throwing activity most because ‘it was calm and I didn’t feel rushed’, whilst another liked the jumping activity most because ‘people were nice and cheering each other’. Although the data provide some valuable generalisations that certainly suggest that effectively delivered competitive challenges can have a positive impact on a class of children, teachers need to consider how this can be delivered appropriately for each child. It is, therefore, proposed that the CLZ could be individualised for the children (Figure 5) who are the least and most confident to facilitate both enjoyment and motor competency development.

#### 4.1.2. The Importance of Children’s Voices

Fun and enjoyment in PE lessons are often ‘by products, rather than direct objectives’ [4] (p. 37) and the researchers stress, therefore, the importance of creating the right environment. Likewise, Beni et al. [17] suggest that ‘fun should not be ignored, nor should it be prioritised at the expense of other criteria for meaningful experiences’ [17] (p. 300). In this study, the results suggest that the competition group enjoyed being set challenges through specific targets, and this type of environment is one that the children overall enjoyed more than the control group who were not set targets and did not enjoy nor perform as well in session 2. Nonetheless, when considering individual scores and the range of qualitative comments in Table 3 and Table 4, the results from this research would suggest that a ‘one size fits’ all approach may not be relevant, particularly when considering the impact of individual perceived competence on performance and enjoyment across the different challenges, as a range of individual target setting is needed for the whole class.

Developing a greater knowledge of the children’s perceptions about the impact of different levels and types of competition may enable educators and practitioners to better understand their pupils’ enjoyment of PE. This could support them in their planning of how and where to use competition effectively in their lessons. A recent review of PE provision at primary level in Irish schools has highlighted the importance of seeking the views and opinions of pupils as one of four key elements to future curriculum design, whereby ‘young people’s experiences and voice has pedagogical relevance and implications’ [16] (p. 217). The qualitative results (in Table 3 and Table 4) illustrate how the children perceive their successes as being a result of tangible and intangible factors. Fewer children focused on external and unstable causes [47] when they had personal targets to focus on for each activity challenge. It was almost as if focusing on the target allowed them to not consider or focus on physicality. There were more examples of physicality, such as ‘it took too much energy’ and it was ‘too exhausting’, as intangible reasons that were shown in the control group. This again suggests that individualised targets set within competing alongside others can positively impact both enjoyment and future motivation in both the activity and PE in general. Likewise, setting lower targets may increase children’s perceived competence and ultimately performance [38] and also help the children feel the sense increased of motor competence that helps make PE lessons more meaningful for children [17]. This may be particularly relevant for children whose perceived competence is already low.

The findings from this research create a number of implications for how teachers may consider planning and delivering competition in PE lessons. Aggerholm et al. [54] offer four ways that future discussions surrounding the use of competition in PE may be considered: ‘avoid, ask, adapt or accept’ (p. 385). The results from this research demonstrate that competition can have a positive impact, even on younger (6–7-year-old) children. Therefore, teachers who avoid the use of competition for fear of the negative connotations associated with de-competition [9] could lead to children actually missing out on the benefits highlighted within this study. Certainly, in the absence of competition in the form of individualised targets, children’s level of competence was lower (and regressed at a higher rate in many cases) (see Figure 2 and Figure 3).

However, teachers who accept the current approach and the emotional impact that winning and losing (in a very traditional sense) have on children could exclude many children from the positive impacts that competition can have [53]. PE lessons that disengage children at a young age could have significant implications on their health and well-being throughout the lifespan [55]. Thus, the findings from this study suggest that teachers need to consider how they might adapt the way that competition is perceived and delivered and the moving of the CLZ within the MELC, which is one proposed method (see Figure 5). If enjoyment is considered one of the major factors that underpin increased physical activity, the results from this research suggest that this group of children enjoyed the challenges more when they were set low targets in a task-involving climate.

Focusing on competing alongside others to improve personal bests would appear to be a good place for children to begin to learn about competition. Helping children to understand and cope with both success and failure and develop the determination and resilience that come from pursuing measurable goals is a worthy learning experience. Competition, in whatever format it comes, however, needs to be delivered in a ‘positive learning climate’ [11] (p. 135). Even when setting low challenges, some children fail to achieve success. This leads to Aggerholm et al.’s fourth consideration: ask [54]. When children in Irish primary schools were asked for their perspectives on what makes their PE lessons meaningful, whilst competition was viewed as meaningful for some and a positive influence on engagement in PE, for others it was not [16].

‘Competition, possibly due to the prevalence of team games, was inextricably linked to motor competence, with those who felt they lacked motor competence reporting to shy away from participation’ [16] (p. 212). This was evident in the range of qualitative comments offered by the pupils in this study where statements, such as ‘I messed up a lot’’, ‘It was too difficult’, and ‘I got worse’, illustrated how, for some, the reasons for enjoying an activity the least relate to low perceived competence (see Table 4).

The challenge for all PE teachers is to create learning opportunities in their PE lessons that engage all students. Trying to achieve lofty goals associated with developing a lifetime passion for physical activity can be particularly challenging when dealing with children whose perception of their own levels of physical competence is low [11]. For these children, participating in competitive activities, particularly those that reinforce their low self-esteem, can be problematic. For some, the ability to choose their level of challenge or even to have the ability to opt-out may foster greater enjoyment of their PE lessons [27]. In this study, the researcher set the level of challenge for each child (albeit based on their individual raw scores from session 1). However, future research might consider the impact of allowing children to choose their own targets.

Fletcher at al. [30] propose that meaningful PE lessons should ensure that learning is personally relevant to each individual. Children who lack confidence and perhaps have lower levels of competence may work best when given lower targets, as these can have a positive impact on their confidence to succeed. For this research, this approach would be particularly relevant for many of the girls. However, for others, a different approach may be required. Teachers need to understand what motivates different individuals and devise individualised approaches (and targets) for each (adjusting the CLZ for the individuals within a class; see Figure 4). Where possible, children should be involved in this process and be given the opportunity to contribute to the process of setting their own targets. These need to be communicated clearly, and constant positive feedback and reinforcement should be offered to children in order to foster greater enjoyment and build greater confidence. This will create a mastery-oriented climate that focuses on each individual’s effort and improvement rather than them necessarily needing to perform with, for, or against others [56].

## 5. Strengths and Limitations

This study is timely, as it contributes to the field of young children’s enjoyment and competency within PE lessons, an under-researched area in the field. This study is the first of its kind to be able to share examples of children’s voices as to why they are enjoying/not enjoying specific target-setting strategies used in PE. The findings could inform future larger studies examining more schools from a wider range of different economic and locational settings. Although more research is needed, another strength of the current study is its novel insight into young children’s points of view; not enough studies have investigated the young age range in this study.

The sample size of participants (n = 97) in this study is a strength. The average size for a single-year group in a primary school in England is 38 pupils [57]. Schools that had a two-form entry system (where there are two classes in each year group) not only enabled the study to have a sample group that was above the national average for each school but it also enabled the researchers to access the control and experiment groups within the same school. This allowed for direct comparison within the same settings. However, the use of only two schools is also acknowledged as a limitation of the research. Likewise, the fact that both the control and competition groups were situated within the same school is a limitation of this study. For future research, upscaling and increasing the number of schools and classes used within the sample would be recommended.

The activities selected for the activity challenges [42] were a strength of this study, as these were developed by the organisation ‘Fit for Sport’ as part of an Activity Challenge programme that was delivered to over 10,000 primary-age children, which provided a benchmark score for each activity, providing strong reliability for the challenges. The benchmark scores have been published as age phases and activity specifics and have a bronze, silver, and gold level. Although these classifications were not applied in this study, future studies could investigate the impact on the enjoyment and perceived competence of children if they were made aware of the particular status of their score in relation to the bronze, silver, and gold standards.

A number of strategies were introduced to foster high levels of internal validity across this study. Ensuring that the sessions took place at the same time on the same days in perfect weather conditions using exactly the same equipment all helped to maintain consistency in the delivery of the activities. However, ensuring test–re-test reliability across different locations and using different staff creates the potential for differences that may skew the results. As highlighted by Scotland [58], ‘often context limits methodology; isolating variables can be difficult’ (p. 11). As much as the reasons for working with children in their own school during their regular PE lesson was an important consideration when designing this study, if it were to be extended to engage a larger sample, some adjustments could be made. For example, bringing the children to one central location that utilises the same staff in an environment that can guarantee a complete replication of the testing conditions for all children would increase the reliability factor.

The challenge of replicating the controlled environment and conditions that investigated the impact of competition on three activities over just two sessions, whilst not being able to control what happened in between, was a limiting factor in this research. Thomas [44] describes how ‘confounding variables’ (p. 172) may influence the results of an experiment. These are often things that happen outside of the testing environment that may still have the potential to skew results. These activities may indirectly influence improvements in competence that are not related to the competition factors that they may be credited within the research. Nonetheless, the results from this small short-term case study that offers comparative results from a very specific geographic and demographic population demonstrate the possible impact that can be achieved with the application of specific individualised approaches to how competition is used. At the very least, the results warrant future research as part of a wider longitudinal study.

## 6. Conclusions

The results from this research demonstrate that competition can have a positive impact, even on younger (6–7-year-old) children. It is recommended that teachers/practitioners working within this age phase should create opportunities for children to compete alongside others by completing competitive challenges (set at an appropriate level for each child) in order to help to raise their perceived competency levels and enjoyment in PE lessons. The idea of a CLZ at the center of the MELC [26] suggests that there is an appropriate level by which competition should be delivered in order to affect the best learning. Children who lack confidence and have a lower perceived competency may work best when given lower targets, so moving the CLZ to the left in the MELC can have a positive impact on their enjoyment of the subject and enhance their perceived competency. This shift in the CLZ also supports the children’s reasoning for their performance to be more tangible rather than intangible, supporting their overall motivation and enjoyment. For other children, a different approach may be required. There is also a need to understand what motivates different individuals by listening to the children’s voices and devising individualised approaches (and targets) for each child (adjusting the CLZ for the class needs). These need to be communicated clearly, and constant positive feedback and reinforcement should be offered to children in order to foster greater enjoyment and build greater perceived competency.

## Figures and Tables

**Table 1:** Activity challenges [42] for session 1.

Category	Challenge
Running	Shuttle run challenge Children run between 2 cones/lines set 6 m apart. The challenge measures how many shuttles children can complete in 1 min.
Jumping	Star jump challenge Children start with their legs together and arms by their side and then jump so both legs and arms go out sideways together (making the shape of a star) before jumping back to their starting shape again. The challenge measures how many star jumps children can complete in 1 min.
Throwing and Catching	Two-handed bounce and catch Using 2 hands and a medium-sized ball, the children bounce a ball on the floor and catch it again whilst standing on one spot. The challenge measures how many bounces and catches children can complete in 1 min.

**Table 2:** Adaptations to the challenges in session 2.

Activity Challenge	Competition Group	Control Group
Running	In session 2, children were set a low target for this activity; 10% less than their individual score from session 1.	In session 2, children were given no specific targets.
	All children competed alongside the same partner as session 1.	All children competed alongside the same partner as session 1.
Jumping	In session 2, children were set a high target for this activity; 10% more than their individual score from session 1.	In session 2, children were given no specific targets.
	All children competed alongside the same partner as session 1.	All children competed alongside the same partner as session 1.
Throwing and Catching	In session 2, children were set a target that was the same score as session 1.	In session 2, children were given no specific targets.
	All children competed alongside the same partner as session 1.	All children competed alongside the same partner as session 1.

**Table 3:** Reasons for most enjoyment.

Competition Group Tangible Responses	Competition Group Intangible Responses	Control Group Tangible Responses	Control Group Intangible Responses
‘Hit Target’	‘Fun’	‘I got the highest score in my group’	‘Good at it’
‘Improvement’	‘Easy’	‘I completed the most runs in the class’	‘Did best in it’
‘High Score’	‘Less tiring than others’		‘I don’t like the others’
	‘Other activities quite boring’		‘People were nice and cheering each other’
	‘Worked Hardest’		‘It made me more confident in my skills’
			‘It was calm and I didn’t feel rushed’

**Table 4:** Reasons for least enjoyment.

Competition Group Tangible Responses	Competition Group Intangible Responses	Control Group Tangible Responses	Control Group Intangible Responses
‘Poor scores’	‘Least good at it’	‘I only got one’	‘It was too difficult’
‘Hard to hit target’	‘Hard work’		‘It was so exhausting’
‘Not able to improve’	‘Too exhausting’		‘It ached me out’
‘I got worse’	‘I messed up a lot’		‘It took too much energy’
	‘It was boring’		‘I was in my plimsoles and I kept slipping over’
			‘It hurt your arms’

## Data Availability

The data presented in this study are available in article.
